# Le lymphœdème congénital primaire: la maladie de Milroy: à propos du premier cas observé dans le Département de Pédiatrie du Centre Hospitalier Universitaire Yalgado Ouédraogo, Ouagadougou

**DOI:** 10.11604/pamj.2017.27.21.11443

**Published:** 2017-05-09

**Authors:** Chantal Zoungrana Ouattara, Angèle Kalmogho, Caroline Yonaba, Chantal Gabrielle Bouda, Ghislaine Yaméogo, Ludovic Kam

**Affiliations:** 1 Département de Pédiatrie du CHU Yalgado Ouédraogo de Ouagadougou, Burkina-faso; 2 Unité de Formation et de Recherche en Sciences de la Santé (UFR/SDS), Université de Ouagadougou, Burkina Faso; 3 Service de Pédiatrie Médicale du CHU Charles de Gaulle de Ouagadougou, Burkina-faso

**Keywords:** Lymphœdème primaire, maladie de Milroy, diagnostic, traitement, Ouagadougou, Primary lymphedema, Milroy disease, diagnosis, treatment, Ouagadougou

## Abstract

Le lymphœdème est l'accumulation de liquide lymphatique dans les espaces interstitiels, celui de l’enfant, la maladie de Milroy, est rare, héréditaire, autosomique dominante à pénétrance partielle. Nous rapportons un cas de maladie de Milroy chez une fillette de 7 ans vue, pour érysipèle sur grosse jambe droite congénitale. Des antécédents de gros membre congénital existent dans la famille maternelle. L’examen retrouvait une grosse jambe droite oedématiée et douloureuse à la palpation, avec une lichenification de la peau en regard et un érysipèle cutané. Le bilan paraclinique objectivait un lymphœdème cutané avec atteinte vasculaire à type d’ectasie de la saphène droite. Le caryotype de type féminin, était sans anomalie, n’excluant pas des remaniements chromosomiques de petite taille. Le traitement a constitué en une kinésithérapie, des bandages, le port de bas de compression et une psychothérapie. Ce premier cas décrit au Burkina Faso témoigne de la rareté de la pathologie mais surtout des difficultés diagnostiques liées à l’insuffisance des investigations paracliniques.

## Abstract (en)

Congenital lymphedema is the accumulation of lymphatic fluid in the child’s interstitial spaces. Milroy disease is a rare, hereditary, autosomal dominant condition showing incomplete penetrance. We report the case of a 7-year old little girl with Milroy disease examined for erysipelas on congenital big right leg. A family history of large congenital member existed. Physical examination showed big oedematous right leg painful to palpation, with skin lichenification and erysipelas. Paraclinical assessment objectified cutaneous lymphedema with vascular involvement suggestive of ectasia of the right saphenous vein. Female karyotype showed no abnormalities, despite the small chromosomal rearrangements. Treatment was based on physiotherapy, bandages, compression stockings and psychotherapy. This first case in Burkina Faso testifies to the rarity of the pathology but especially to the diagnostic difficulties related to the inadequacy of paraclinical investigations.

## Introduction

Le lymphœdème se définit comme une accumulation de liquide lymphatique dans les espaces interstitiels, lié à un dysfonctionnement du système lymphatique. Il touche de manière élective le membre inférieur; on distingue deux formes: primaire et secondaire (filariose, cancer du sein). La maladie de Milroy est un lymphœdème congénital primaire. Son incidence est estimée à moins de un cas pour 100 000 naissances; c’est une maladie héréditaire, autosomique dominante à pénétrance partielle, atteignant surtout les filles [1–3]. C´est une pathologie méconnue des praticiens dans nos contrées.

## Patient et observation

**Interrogatoire:** OA, fillette de 7ans, a consulté pour une éruption cutanée prurigineuse d’apparition récente, siégeant sur une grosse jambe droite congénitale. La symptomatologie remontait à une semaine avant la consultation, par une éruption cutanée sur le tiers inférieur de la jambe droite, éruption prurigineuse s’excoriant par le grattage, le tout sur un fond douloureux

**Figure 1:**
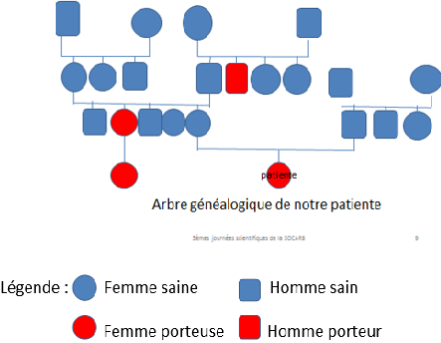
Arbre généalogique de notre patiente

**Antécédents:** la fillette est issue d’une grossesse bien suivie, avec un accouchement eutocique. A la naissance, il est constaté une inégalité de grosseur des membres: la jambe droite et le bras gauche apparaissant plus gros que leurs controlatérales. Elle avait bénéficié de divers traitements médicamenteux sans succès. L’évolution s’est faite vers la normalisation du volume du bras gauche vers l’âge de 4 ans. Par contre la jambe droite augmenta lentement de volume, la peau en regard devenant plus épaisse avec des lésions papulo-prurigineuses apparaissant par intermittence. Sur le plan familial, il existait une notion de gros membre congénital chez certains parents (Figure 1).

**Figure 2:**
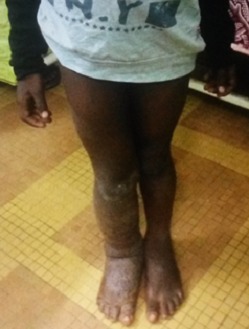
Aspect boudiné du membre pelvien droit

**Figure 3:**
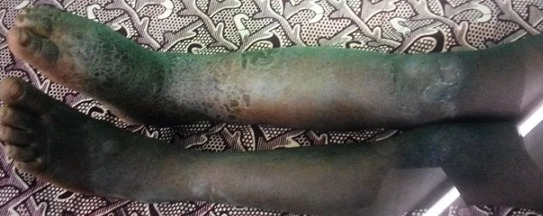
Erysipèle siégeant au tiers inférieur de la jambe

**Examen physique:** 1) un bon état général, les constantes: poids: 21 kg; Taille: 120 cm; IMC: 14,58 kg/m^2^; T°: 37,4 C, TA: 100/70 au bras droit et 100/60 au bras gauche; 2) Examen des membres inférieurs notait: une jambe droite plus grosse (Tableau 1): le membre pelvien droit paraît boudiné, de même longueur que la controlatérale. Une douleur au membre inférieur droit cotée à 5/10, avec une limitation de la flexion dorsale, rotation interne et externe du pied; la force musculaire est mesurée à 4/5 au quadriceps et au mollet droit, la marche est possible (Figure 2). Le tiers inférieur de la jambe droite et le dos du pied droit avait un aspect lichenifié, avec la peau en regard épaisse et cartonnée, le tiers inférieur de la jambe droite et les malléoles présentaient un érysipèle (Figure 3); 3) les autres appareils ne présentaient pas d´anomalie.

**Tableau 1:** Circonférence des membres pelviens prise jambe en extension à l’admission

Circonférence	Membre pelvien droit à l’admission	Membre pelvien droit à la phase de stabilisation
Trochantérien	47	**45**
Mi-cuisse	38	**36**
Rotule	31	**29**
Jambe : 1/3 sup- 2/3 inf	26	**25**
Jambe : 2/3 sup- 1/3 inf	21	**19**
Bi-malléolaire	24	**23**
Pli métatarso-phalangien	23	**21**
Signe de Stemmer	Positif	**Positif**

**Figure 4:**
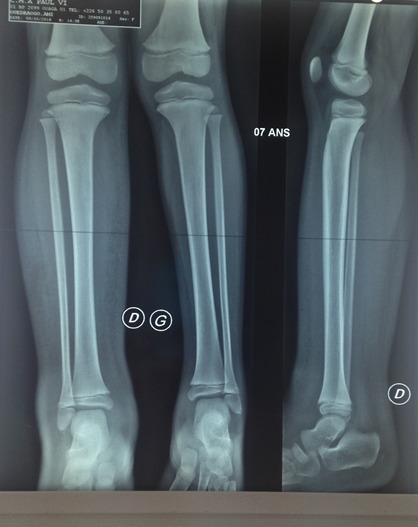
Rx comparative des 2 jambes

**Figure 5:**
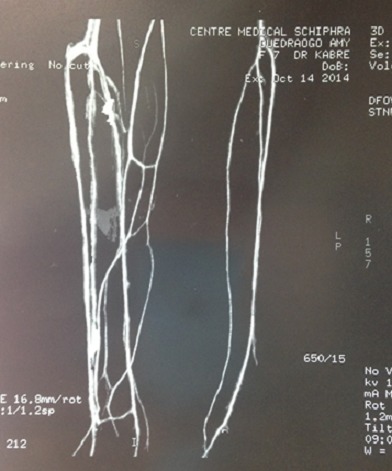
Angioscanner des jambes

**Examens complémentaires:** 1) la radiographie standard notait un gonflement des parties molles au niveau de la cuisse, de la jambe et du dos du pied droit comparativement au membre pelvien controlatéral (Figure 4); 2) sur l’angioscanner, on notait un réseau vasculaire plus abondant au membre inférieur droit témoin d’une néo vascularisation cependant sans obstacles (Figure 5); 3) à l’échographie Doppler du membre inférieur droit, on notait ectasie veineuse de la grande saphène et de ses branches superficielles ; lymphœdème de la cuisse, jambe et cheville: troubles trophiques avec épaississement du derme et du sous derme et des parties molles; 4) Les échographies cardiaque et abdominale étaient normales; 5) la biologie: la biologie moléculaire: le caryotype est féminin sans anomalies (dans les limites des techniques utilisées), n’excluant pas les remaniements chromosomiques de petite taille. Une étude complémentaire du caryotype sanguin par puce à ADN a été proposée. Les numérations sanguines, vitesse de sédimentation, protidémie, et créatininémie étaient normales, l´électrophorèse de l’hémoglobine était AA.

**Traitement:** symptomatique avait pour buts de soulager la douleur, réduire et stabiliser le volume du membre, améliorer la fonction du membre et la qualité de vie, prévenir les complications Notre patiente a bénéficié d’un traitement à base d’antibiotiques et de soins cutanés avec topiques locaux, d’un drainage lymphatique manuel par kinésithérapie, de bandages multicouches puis compression élastique par bas de compression et d´une psychothérapie

**Evolution:** favorable avec une guérison de l’érysipèle, la fonction du membre améliorée et la douleur atténuée ainsi qu’une légère diminution du membre pelvien droit.

## Discussion

Notre patiente était de sexe féminin et avait 7 ans au moment du diagnostic. Smeltzer et al. en 1985, notaient une incidence annuelle française du lymphœdème primaire congénital de 11,5 cas par million d’habitants (-20 ans) avec une nette prédominance féminine [4] et Baulieu et al. un sex-ratio de 1, avec un âge moyen au diagnostic de 9 ans; ¼ des cas avant 2 ans [5]. La maladie de Milroy est une maladie génétique autosomique dominante à pénétrance partielle avec une incidence de moins de un pour 100 000 naissances [1–3]. Chez notre patiente, les antécédents familiaux du côté maternel et le lymphœdème présent à la naissance, témoignent de son caractère familial et héréditaire. Baulieu et al. dans leur étude notaient que 4 enfants avaient des antécédents familiaux [5, 6]. La littérature rapporte cependant des cas sporadiques liés à des mutations génétiques de novo [2, 7–9].

La fonction de marche du membre était conservée comme dans les études de Carver et al. Kitsiou-Treli [6, 7]. Baulieu, notait une impotence fonctionnelle relative dans le groupe des adolescents [5]. Le jeune âge de notre enfant peut l´expliquer et il est à craindre cette éventualité au fil des années. Nous avons retrouvé un érysipèle et les complications infectieuses sont décrites dans la littérature allant de l’érysipèle, les pleuro-pneumopathies, les infections digestives et les myocardites [5, 7, 10]. Le lymphœdème a quasiment été confirmée par l’échographie doppler comme pour la plupart des autres auteurs [3, 5, 7, 10]. Notre patiente avait une ectasie veineuse avec une néo vascularisation, Baulieu retrouvait 7 patients porteurs de malformations vasculaires; pour Salfini, une insuffisance veineuse était associée au lymphœdème [11]. Des malformations vasculaires peuvent être associées [5, 9]. La lymphographie n’a pas été faite; elle est cependant d’un apport certain pour le diagnostic. La biologie moléculaire notait un caryotype féminin sans anomalie décelée dans la limite des techniques utilisées elle n’exclut pas les remaniements chromosomiques de petite taille La localisation génique du lymphœdème est en 5q 35-3. Cette localisation suggère un lien avec le gène du vascularendothelialgrowth factor: VEGF [8].

Le traitement a été symptomatique. La littérature rapporte l’intérêt des topiques locaux et des macrolides dans le traitement des complications cutanées [10]. Puis le traitement du lymphœdème qui s’est poursuivi par un drainage lymphatique manuel et bandage multicouches (puis compression élastique par bas de compression), sont décrits dans la littérature [4, 5, 7]. L’évolution chez notre patiente a été favorable, marquée par une diminution puis une stabilisation de l’œdème. Baulieu, Carver, Smeltzer et al. ont rapporté également une stabilisation voire une régression complète de l’œdème avec l’âge [4, 5, 7]. Cependant on peut avoir une réapparition à la faveur d’un traumatisme ou d’une grossesse chez la fille [2].

## Conclusion

La maladie de Milroy est une maladie héréditaire autosomique dominante à pénétrance partielle dans la plupart des cas. Elle est facile à reconnaitre mais le diagnostic positif, basé sur la biologie moléculaire, nécessite des investigations onéreuses pour un pays à faibles revenus. Son traitement est symptomatique nécessitant un suivi régulier tout au long de la vie pour prévenir les complications.

## Conflits d’intérêts

Les auteurs ne déclarent aucun conflit d'intérêts.
